# Encapsulation of Phloroglucinol from *Rosenvingea intricata* Macroalgae with Zinc Oxide Nanoparticles against A549 Lung Cancer Cells

**DOI:** 10.3390/pharmaceutics16101300

**Published:** 2024-10-05

**Authors:** Sakthivel Muthu, Mythileeswari Lakshmikanthan, Edwin Edward-Sam, Mutheeswaran Subramanian, Lakshmanan Govindan, Afrina Begum Mithen Patcha, Kathiravan Krishnan, Nallusamy Duraisamy, Selvakumari Jeyaperumal, Al Thabiani Aziz

**Affiliations:** 1 Center for Global Health Research, Saveetha Medical College and Hospital, Saveetha Institute of Medical and Technical Sciences (SIMATS), Thandalam, Chennai 602105, Tamil Nadu, India; 2 Department of Biotechnology, University of Madras, Guindy Campus, Chennai 600025, Tamil Nadu, India; mythileeswari@gmail.com (M.L.); afrinabegum1121@gmail.com (A.B.M.P.); 3 Department of Microbiology, Division of Virology and Molecular Biology, St. Peters Medical College Hospital & Research Institute, Hosur 635130, Tamil Nadu, India; edwin280@gmail.com; 4 Xavier Research Foundation, St. Xavier's College, Palayamkottai, Tirunelveli 627002, Tamil Nadu, India; muthees2009@gmail.com; 5 Department of Anatomy, Saveetha Medical College and Hospital, Saveetha Institute of Medical and Technical Sciences (SIMATS), Thandalam, Chennai 602105, Tamil Nadu, India; lakshmanang261988@gmail.com; 6 Department of Research, Meenakshi Academy of Higher Education and Research (MAHER), Chennai 600078, Tamil Nadu, India; nallfree07@gmail.com; 7 National Centre for Disease Control, Thiruvananthapuram Field Unit, Iranimuttam, Thiruvananthapuram 695009, Kerala, India; jselvakumari87@gmail.com; 8 Department of Biology, Faculty of Science, University of Tabuk, Tabuk 71491, Saudi Arabia; aalthbyani@ut.edu.sa; 9 Biodiversity Genomics Unit, Faculty of Science, University of Tabuk, Tabuk 71491, Saudi Arabia

**Keywords:** phloroglucinol, *Rosenvingea intricata* algae, ZnO-PEG-PHL NPs, antioxidant assays, anticancer effects

## Abstract

Background/Objectives: Phloroglucinol (PHL), a phenolic compound extracted from the brown alga *Rosenvingea intricata*, exhibits potent antioxidant and anticancer properties. This study aims to extract, purify, and characterize PHL, and further develop functionalized zinc oxide nanoparticles (ZnO NPs) loaded with PHL to enhance its therapeutic potential. Methods: PHL was extracted using acetone and purified through Sephadex LH-20 column chromatography, yielding a highly enriched fraction (F-3). The purified compound was characterized by FTIR, HPLC, NMR, and LC-MS. ZnO NPs were synthesized, PEGylated, and conjugated with PHL, forming ZnO-PEG-PHL NPs. Their characterization included DLS, zeta potential, XRD, SEM-EDAX, and encapsulation efficiency studies. Antioxidant assays (DPPH, FRAP, ABTS, RPA) were performed and in vitro cytotoxicity on A549 lung cancer cells were determined to evaluate the therapeutic efficacy of PHL. Results: The purified PHL fraction showed a high phenolic content (45.65 PHL mg/g), which was was confirmed by spectral analysis. The ZnO-PEG-PHL NPs increased in size from 32.36 nm to 46.68 nm, with their zeta potential shifting from −37.87 mV to −26.82 mV. The antioxidant activity was superior for the ZnO-PEG-PHL NPs in all assays, while the in vitro cytotoxicity tests showed an IC_50_ of 40 µg/mL compared to 60 µg/mL for the ZnO NPs and 70 µg/mL for PHL. Apoptotic studies revealed significant cell cycle arrest and apoptosis induction. Conclusions: The synthesized ZnO-PEG-PHL NPs demonstrated enhanced antioxidant and anticancer properties, making them promising candidates for cancer therapy and antioxidant applications.

## 1. Introduction

Natural products obtained from brown seaweeds have attracted considerable interest recently due to their potential anticancer effects. These marine organisms are abundant in bioactive compounds such as polysaccharides, notably sulfated polysaccharides [1], phlorotannins [2], carotenoids [3], and polyphenols [4], all of which have demonstrated significant cytotoxic and anti-proliferative activities against diverse cancer cell lines [5,6]. The unique structural features of these compounds, such as the sulfate groups in fucoidan and multiple hydroxyl groups in phlorotannins, contribute to their ability to modulate key signaling pathways involved in cancer progression, such as apoptosis, cell cycle arrest, and metastasis [7,8]. Furthermore, the antioxidant activity of these components helps in mitigating oxidative stress, a known factor in cancer development, thus making brown seaweeds a valuable source of natural anticancer agents [9,10]. Phlorotannins are polyphenolic compounds that are present in brown seaweeds, distinguished by their polymeric structures composed of phloroglucinol units. These compounds demonstrate notable antioxidant, anti-inflammatory, and anticancer properties, which are ascribed to their capacity to neutralize free radicals and influence various cellular signaling pathways [11,12].

Lung cancer remains a predominant cause of cancer-related mortality globally, impacting populations across both industrialized and less-developed nations. The therapeutic approaches for lung cancer are diverse, including chemotherapy, biological treatments such as immunotherapy, and innovative techniques like nanoparticle-mediated targeted drug delivery [13,14]. NPs offer a promising therapeutic avenue by enabling the targeted delivery of drugs to cancer cells, thereby minimizing the damage to normal tissues and reducing side effects [15,16]. Zinc oxide nanoparticles (ZnO NPs) show significant potential in inducing oxidative stress within cancer cells [17,18,19]. To improve the therapeutic efficacy and bioavailability of phloroglucinol (PHL), it has been incorporated into NPs, particularly ZnO NPs. Functionalizing ZnO NPs with polyethylene glycol (PEG) and incorporating PHL—a polyphenolic compound with established anticancer effects—can increase the stability and bioavailability of these NPs [20,21]. 

Existing research highlights the anticancer potential of bioactive compounds found in brown algae. Isolating PHL from these seaweeds requires multiple purification steps, starting with biomass collection and drying. Their extraction is typically performed using organic solvents such as ethanol or methanol, which are effective in isolating polyphenolic compounds [22,23,24]. Purification is crucial for obtaining high-purity phloroglucinol (PHL) post-extraction, and is typically done using column chromatography [25,26]. PHL and its derivatives have been identified as natural compounds from plants, algae, and microbes, and have been shown to have antioxidant, antibacterial, anticancer, anthelminthic, and antidiabetic properties [22]. The purified PHL is then utilized to fabricate NPs ranging from 1 to 100 nm. ZnO NPs are widely employed in biomedical fields due to their biocompatibility, minimal toxicity, and photocatalytic properties [27,28,29]. Various synthesis methods, such as the sol-gel [30,31], precipitation [32,33], hydrothermal [34,35], and microwave-assisted techniques [36,37], are utilized for the fabrication of ZnO NPs. The sol-gel method is particularly advantageous for controlling the particle size, morphology, and surface properties [38].

The synthesis of ZnO NPs involves reacting zinc salts with appropriate precursors under controlled conditions, followed by annealing to achieve the desired crystalline structure [36,37]. The surface modification of ZnO NPs through PEGylation improves their stability and biocompatibility. PEGylation, which involves the covalent attachment of PEG chains to nanoparticle surfaces, reduces immune system recognition and enhances the NPs’ ability to evade clearance mechanisms, thereby extending their circulation time in the bloodstream [39,40,41]. Gold NPs and ZnO NPs that were synthesized and conjugated with PHL were evaluated as potential antibiofilm and antivirulence agents [20].

Conjugating PHL with ZnO NPs through PEGylation (ZnO-PEG-PHL NPs) represents a synergistic strategy for cancer therapy [20]. PHL exerts anticancer effects through various mechanisms, including inducing apoptosis, inhibiting cell proliferation, and modulating cancer-related signaling pathways [42]. When encapsulated within ZnO NPs and PEGylated for enhanced stability and biocompatibility, PHL can be selectively delivered to lung cancer cells, minimizing its systemic toxicity and maximizing its therapeutic efficacy. By integrating these technologies, this research aims to advance therapeutic strategies against lung cancer using natural compounds and nanotechnology, contributing to the evolution of cancer treatment modalities.

This research investigates the extraction and purification of PHL from brown seaweeds, followed by the synthesizing of ZnO NPs. These ZnO NPs are subsequently functionalized with polyethylene glycol (PEG) and PHL, resulting in ZnO-PEG-PHL NPs. This study further evaluates the in vitro drug release profiles of the NPs, assesses their antioxidant activities, performs cytotoxicity assays, and examines the therapeutic efficacy of the NPs against A549 lung cancer cells.

## 2. Materials and Methods

### 2.1. Collection of Seaweeds

Fresh specimens of *R. intricata* were gathered from the coastal area of Mandapam in the Gulf of Mannar (latitude 8°35′ N to 9°25′ N and longitude 78°08′ E to 79°30′ E). The seaweeds were first rinsed thoroughly with seawater to eliminate surface debris and epiphytes [43]. Subsequently, the biomass underwent multiple washes with tap water followed by a final rinse with distilled water [44,45].

### 2.2. Extraction of Crude and Partial Purification of PHL

Fresh specimens of *R. intricata* were obtained and identified taxonomically following established reference methods [46]. The algae were carefully desiccated and pulverized into powder. The powdered material was subsequently stored at 4 °C for subsequent analysis. Polyphenolic compounds were extracted using a modified protocol outlined by Li et al. [26]. 100 g of powdered seaweed was subjected to defatting with ethyl acetate. This was followed by extraction with a methanol–chloroform mixture (7:2 *v*/*v*), stirred and then centrifuged at 10,000 rpm for 20 min. The supernatant was mixed with acetone and water in a 7:3 volume ratio, stirred for 20 min, and subsequently centrifuged at 10,000 rpm for 1 h. The resulting supernatant was collected and preserved for subsequent analysis. This crude extract is suitable for assessment of its total phenolic and phlorotannin content.

The supernatant was concentrated using rotary evaporation at 50 °C for 2 h. The partially purified phlorotannin (PHL) extract was then lyophilized and stored at room temperature. The total phenolic and total phlorotannin contents of this compound were then assessed.

### 2.3. Purification of PHL

Lyophilized, partially purified PHL underwent chromatographic separation using a Sephadex LH-20 column (Catalog No: LH20100; Sigma-Aldrich, Bangaluru, India). A slurry was prepared by mixing methanol and hexane (60% and 20% *v*/*v*) with the resin, followed by incubation for 2 h to activate it. The activated slurry was then loaded into a column (25.5 cm in length, 2.5 cm in width, with a matrix filling length of 18.5 cm) and equilibrated with methanol. The sample was subsequently loaded into the column. Chromatographic separation involved fractionating the sample into five fractions (from F-1 to F-5) using the following elution solvents: F-1 (50% methanol), F-2 (75% methanol), F-3 (100% methanol), F-4 (methanol/acetone, 5:1 *v*/*v*), and F-5 (methanol/acetone, 3:1 *v*/*v*), as described by Wang et al. [47]. Each fraction was analyzed for total phenolics [48] and phlorotannin contents [47]. The active fractions were combined, concentrated using rotary evaporated at 50 °C for 90 min, and then lyophilized for subsequent studies.

### 2.4. Estimation of Total Phenolic and Phlorotannin Content

The total phenolic contents of crude, partially purified, and purified PHL from *R. intricata* were determined using a modified Folin–Ciocalteu assay as per Farasat et al. [48]. In this method, 20 µL of each extract was mixed with 100 µl of Folin–Ciocalteu reagent (diluted 1:10) and 80 µL of 7.5% (w/v) Na_2_CO_3_. After incubating in the dark for 2 h at room temperature, absorbance was measured at 600 nm. Gallic acid served as the standard, with TPC values expressed in mg GAE g^−1^ of dried extract and fresh weight.

The total phlorotannin content was measured using a modified version of Wang et al.’s [47] method. A 1.0 mL sample (0.1–8 mg/mL) was mixed with 5 ml of 10% Folin–Ciocalteu reagent. After a 5 min reaction, 4 mL of 7.5% Na_2_CO_3_ solution was added. Following a 2 h incubation in the dark at room temperature, absorbance was measured at 725 nm using a Shimadzu UV-1601 spectrophotometer. Calibration curves with PHL solutions (20–100 μg/mL) yielded results as grams of PHL equivalents 100 g^−1^ of extract.

### 2.5. Instrumental Characterization of Purified PHL

The characterization of PHL extracted from *R. intricata* was conducted utilizing various analytical techniques. Ultraviolet-visible absorption spectra were recorded with a Shimadzu 1800 series UV-Visible spectrophotometer, employing 70% acetone as the blank solution, as per the methodology of Sathya et al. [49]. FTIR spectroscopy was conducted on a Thermo Fisher Scientific NICOLET iS50 FT-IR instrument, outlined by Karthik et al. [50]. HPLC-RID was carried out by an Agilent 1200 series system with a C18 column and UV detector, with ethanol as the eluent and a gradient increase of 4% per minute, and with fraction collection as described by Karthik et al. [50]. NMR spectroscopy (^1^H and ^13^C) was conducted on a 500 MHz FT NMR spectrometer, utilizing the acetone as an internal reference standard. Mass spectrometry study was carried out with a Shimadzu Prominence-i series PDA Detector coupled with an LCMS-8040 triple quadrupole mass spectrometer, following the procedure detailed by Choi et al. [51]. The LC-MS system utilized a Hysil C18 column for separation, employing a gradient elution method with water, methanol, and formic acid as the mobile phases. Analysis was carried forward at a column temp. of 40 °C, with PHL samples prepared in a water–methanol diluent (9:1) and injected at a volume of 20 µL with a flow rate of 1.0 mL/min over a 15 min runtime. The electrospray ionization (ESI) interface facilitated analyte ionization for mass spectrometric detection, while the photodiode srray (PDA) detector provided additional spectral data for compound identification and quantification, enhancing the system’s overall analytical capabilities [52].

### 2.6. Synthesis of ZnO NPs

In the research conducted by Akpomie et al. [53], ZnO NPs were synthesized via a one-pot chemical precipitation method. Initially, 4.0 g of zinc acetate was dissolved in 100 mL of distilled water, and the solution was stirred at 60 °C for 40 min using a magnetic stirrer and adjusted to pH 11.0 by adding 0.2 M NaOH, leading to the development of a white precipitate consisting of ZnO and Zn(OH)_2_. This mixture was continuously stirred for an extra hour and then left to rest for 50 min. The precipitate was then filtered and rinsed repeatedly with double-distilled water until achieving a neutral pH, followed by an ethanol wash. The precipitate was later subjected to thermal drying at 80 °C in an oven for 1 h to convert any residual Zn(OH)_2_ to ZnO NPs, and this product was utilized for subsequent research.

### 2.7. The Linking of ZnO NPs and PHL Facilitated by PEG

A 4 mM solution of PEG was prepared by dissolving PEG in 40 mL of double-distilled water, followed by stirring for 2–5 h at room temperature. Subsequently, 20 mL of this PEG solution was added to 10 mL of a suspension containing 0.1 g of chemically synthesized ZnO NPs, and the mixture was stirred at 60 °C for 4 h. This was followed by sonication at the same temperature for 1 hour to ensure uniform coating of the ZnO NPs with PEG. The PEG-coated NPs were then separated via centrifugation at 10,000 rpm for 20 min to remove any uncoated material. For the preparation of ZnO-PEG-PHL NPs, 50 mL of a 10 mg/mL PHL solution was combined with the PEG-coated ZnO NPs, and the process was repeated under the same conditions as described earlier. The final ZnO-PEG-PHL nanoconjugates were then dried and stored for subsequent use, following the procedure described by Ibne Shoukani et al. [54].

### 2.8. Instrumental Characterization of ZnO NPs and ZnO-PEG-PHL NPs

The ZnO NPs and ZnO-PEG-PHL NPs were subjected to extensive characterization using a range of analytical techniques. Ultraviolet-visible absorption spectroscopy was employed to acquire absorption spectra from 200 to 800 nm, following the protocol established by Albarakaty et al. [55]. The stability of the ZnO NPs and ZnO-PEG-PHL NPs was monitored over 30 days with measurements taken at intervals of 1, 5, 10, 15, 20, 25, and 30 days. These measurements were performed using a Shimadzu UV-1601 spectrophotometer (Japan), with the samples prepared in quartz cuvettes and diluted as necessary. FTIR spectroscopy was conducted over the range of 4000–400 cm^−1^, adhering to the methodology described by Albarakaty et al. [55]. DLS and zeta sizer measurements were performed at 25 °C with a 90° scattering angle using a HORIBA Scientific NanoPartica SZ-100 analyzer, with the NPs dispersed in deionized water. XRD patterns were obtained using a Bruker D8 Advance X-ray diffractometer equipped with Cu Kα radiation, as outlined by Al-Dhabi et al. [56]. The scans covered a 2θ range from 5° to 90°, with a scan rate of 1° (2θ) per minute. NP size was determined using the Debye–Scherrer equation. SEM was carried out using a FEI Quanta FEG 200 HR-SEM, which features a customizable graphical user interface and an auto coater for sample preparation, as described by Mohan and Renjanadevi [57].

### 2.9. EE and LC of ZnO-PEG-PHL NPs

To evaluate the encapsulation efficiency (EE) and loading capacity (LC) of ZnO-PEG-PHL NPs, 20 mg of the synthesized PEGylated ZnO NPs were dispersed in 100 mL of DDH_2_O. The dispersion was stirred at 1200 rpm for 20 min at room temperature, explained by Ibne Shoukani et al. [54]. PHL was added to the nanosuspension in varying concentrations of 2.5, 5, 10, 20, and 40 mg/mL. To determine the encapsulation efficiency, the nanosuspensions were centrifuged for 20 minutes at 10,000 rpm and a temperature of 4 °C. The PHL concentration in the supernatant was quantified spectrophotometrically at 720 nm using a Shimadzu UV-1601 spectrophotometer (Kyoto, Japan). EE and LC were calculated using the following formulas:

EE (%) = (Total drug − Free drug/Total drug) × 100

LC (%) = (Total drug − Free drug/Nanoparticle weight) × 100

These formulas quantify the efficiency of drug encapsulation within the NPs and the capacity of the NPs to carry the drug, respectively.

### 2.10. In Vitro Drug Release Study

The PHL, ZnO NPs, and ZnO-PEG-PHL NPs were evaluated using a modified protocol based on Chao et al. [58]. For this assessment, PHL, ZnO NPs, and ZnO-PEG-PHL NPs were dialyzed using membranes with a molecular weight cutoff of 14 kDa and placed in 500 mL volumetric flasks filled with phosphate-buffered saline (PBS) at pH 5.4 and 7.4. The release system was continuously stirred at 200 rpm to maintain homogeneity. At predefined intervals (0, 1, 3, 6, 9, 12, 15, 18, 21, 24, 27, 30, 33, and 36 h), 1.0 mL samples of the release medium were removed and substituted with an equal volume of fresh PBS at the same pH. The absorbance of PHL and ZnO NPs samples was quantified at 350 nm and 720 nm, respectively, utilizing ultraviolet-visible absorption spectroscopy (Shimadzu UV-1601, Kyoto, Japan). Each experiment was performed three times, and the results shown are the mean to ensure accuracy.

### 2.11. Antioxidant Activities

The antioxidant capacities of purified PHL extracted from *R. intricata*, synthesized ZnO NPs, and ZnO-PEG-PHL NP conjugates were evaluated using various assays: the DPPH radical scavenging assay, the ferric reducing antioxidant power (FRAP) assay, the 2,2′-azino-bis-3-ethylbenzothiazoline-6-sulfonic acid (ABTS) assay, and the reducing power assay (RPA). In the DPPH assay, adapted with minor modifications from Sathya et al. [49], a 0.1 mM DPPH solution in methanol was mixed with the test samples in equal volumes and incubated in the dark. For the FRAP assay, according to the procedure of Ummat et al. [59], a preheated FRAP reagent was combined with the samples, and absorbance was measured at 593 nm. The ABTS assay, based on Sunarwidhi et al. [60], involved using a freshly prepared ABTS solution from stock, and OD @ 734 nm after incubation with the samples. In the RPA, as described by Chouh et al. [61], PHL and ZnO-PEG-PHL NP samples were mixed with phosphate buffer and potassium ferricyanide (K_3_Fe(CN)_6_), incubated, and OD @ 700 nm (Shimadzu UV-1601, Kyoto, Japan) following the addition of trichloroacetic acid (TCA) and ferric chloride (FeCl_3_). Ascorbic acid was used as a standard for each antioxidant activity assay.

### 2.12. Cell Culture Maintance and Cell Viability Assay

The A549 lung adenocarcinoma cell line (NCCS, Pune) was cultured in DMEM with 10% fetal bovine serum (FBS) and 1% antibiotic-antimycotic solution. Cells were incubated at 37 °C in a 5% CO_2_ humidified atmosphere and sub-cultured at confluence. For long-term storage, cells were cryopreserved

The MTT assay was used to evaluate cell viabilities of PHL, ZnO NPs, and ZnO-PEG-PHL NPs. Cells were seeded at 6 × 10^3^ cells/well in 96-well plates and treated with substances (10–100 µg/mL in PBS). After 48 h of incubation at 37 °C with 5% CO_2_, DMEM was added, and cells were exposed to MTT solution for 4 h. Formazan crystals were dissolved in DMSO, and absorbance was measured spectrophotometrically. Cell viability was calculated as a percentage of untreated controls using the formula:

Cell viability (%) = (OD Sample/OD Control) × 100.

### 2.13. Apoptosis Assay

This study assessed the apoptosis effects of PHL, ZnO NPs, and ZnO-PEG-PHL NPs using acridine orange/ethidium bromide (AO/EtBr) staining, with modifications from Stankovic et al. [62]. A549 cells were plated at 4 × 10^4^ cells per well in DMEM and incubated at 37 °C with 5% CO_2_. After reaching confluence, cells were treated with different concentrations of PHL, ZnO NPs, and ZnO-PEG-PHL NPs for 48 h, with non-treated cells as controls. Post-treatment, cells were stained with AO/EtBr and analyzed for apoptosis using fluorescence microscopy with green and red filters.

### 2.14. Cell Cycle Distribution Analysis

For cell cycle distribution analysis, A549 cells (4 × 10^6^) were seeded into 6-well plates and treated with PHL, ZnO NPs, and ZnO-PEG-PHL NPs for 24 h. Post-treatment, cells were harvested, washed with phosphate-buffered saline (PBS), and fixed in 70% ethanol at 4 °C. After fixation, cells were stained with propidium iodide (PI) containing RNase and analyzed using flow cytometry to determine the distribution of cells across the G0/G1, S, and G2/M phases of the cell cycle. Data were processed using FlowJo software to quantify the percentage of cells in each phase and assess the impact of the treatments on cell cycle progression.

## 3. Results and Discussion

### 3.1. Extraction of Crude and Partial Purification of PHL

Pulverized algal powder from *R. intricata* was subjected to extraction using an acetone solvent system. The resulting extract exhibited substantial yields and was notably rich in phenolic and phlorotannin compounds, with measurements of 18.09 GAE mg/g and 24.09 PHL mg/g, respectively (Table 1). Following the extraction, the acetone was removed from the crude extract through rotary evaporation. The partially purified extract, referred to as PHL, was then analyzed to quantify its phlorotannin content. The results indicated that its total phenolic content was 21.03 GAE mg/g and its total phlorotannin content was 30.91 PHL mg/g (Table 1). When comparing these results to the findings of Dang et al. [63], significant discrepancies in the total phenolic and phlorotannin contents were noted, as they obtained total phenolic content values of 12.83 GAE mg/g, 14.29 GAE mg/g, and 26.56 GAE mg/g for crude, partially purified, and purified extracts, respectively. Additionally, they observed total phlorotannin contents of 4.7 PGE mg/g in crude, 0.4 PGE mg/g in partially purified, and 9.4 PGE mg/g in purified extracts [63]. These differences could be attributed to variations in extraction methodologies, such as the choice of solvent, extraction conditions, and sample preparation techniques. Furthermore, differences in algal species, geographical locations, and environmental factors could also account for the observed variations in phenolic and phlorotannin content across studies.

### 3.2. Purification of PHL

**Figure 1:**
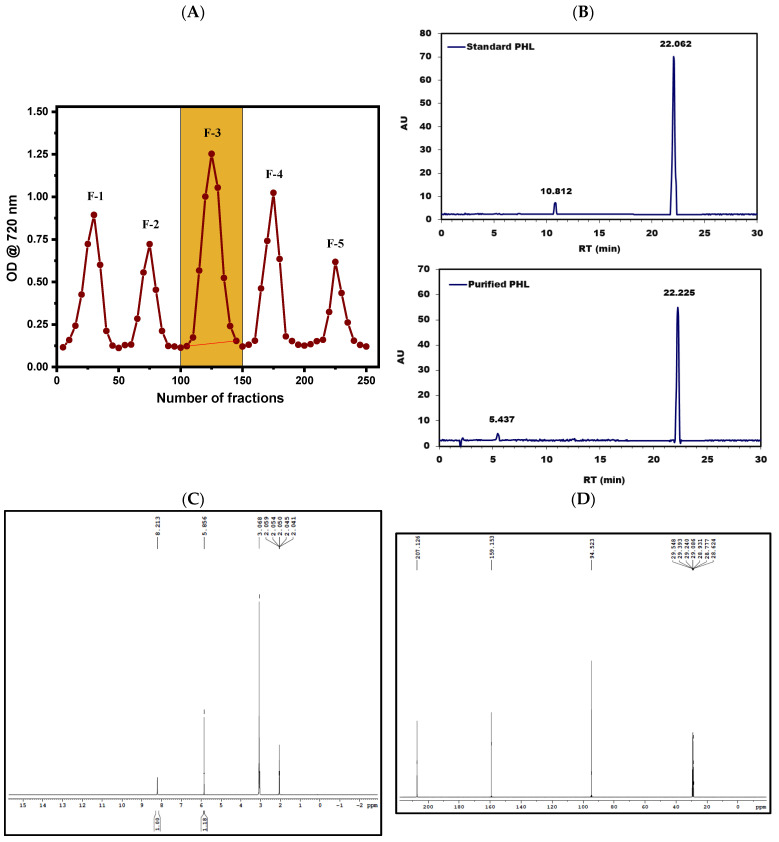
Sephadex LH-20 column chromatography was used to elute PHL from *R. intricata*, yielding fractions designated as F-1, F-2, F-3, F-4, and F-5 (**A**). HPLC chromatograms of the purified PHL and standard PHL were shown in (**B**). Purified PHL was further analyzed using ^1^H and ^13^C NMR, as depicted in (**C**) and (**D**), respectively.

A partially purified PHL sample was dissolved in ethanol at an amount of 50 mg/5 mL and subjected to Sephadex LH-20 chromatography. Then, it was eluted using a range of solvent gradients, labeled as F-1 through F-5. The yield of each fraction was measured, and the absorbance of each was recorded. These fractions were then pooled separately based on the solvent gradient employed. The F-3 fraction (from fractions 100 to 150), which was eluted with pure methanol, demonstrated notably high phenolic and phlorotannin contents, quantified at 32.47 GAE mg/g and 45.65 PHL mg/g, respectively. This F-3 fraction was selected for further investigation in subsequent studies (Figure 1A and Table 1). A comparison with the results by Wang et al. [64] shows discrepancies in total phlorotannin contents between studies. Compared to our findings, Wang et al. [64] reported a slightly higher total phlorotannin content of 23.21 PGE mg/g. These differences could be attributed to variations in extraction methods, the chromatographic conditions, or the characteristics of the samples. Other contributing factors could include the specific species of brown algae used, the geographical location, or environmental conditions that may impact the phlorotannin content. It is crucial to highlight the disparity in total phenolic content found between our research and the study conducted by Wang et al. [64]. While our eluted fractions had a total phenolic content of 32.47 GAE mg/g, Wang et al. did not provide comparable data for total phenolic content. This highlights the necessity of thorough analysis and the comprehensive reporting of bioactive compounds during purification processes to enable the accurate comparison and interpretation of results.

### 3.3. Instrumental Characterization of Purified PHL

#### 3.3.1. FTIR Spectroscopic Analysis

FTIR spectroscopy of the PHL revealed key details about its molecular structure through various absorption bands. A strong peak at 3200 cm^−1^ indicated the stretching vibration of hydroxyl (O-H) groups, confirming the presence of alcohol functionalities. The band at 1619 cm^−1^ corresponded to C-O stretching, suggesting ester groups in the molecule, consistent with PHL’s ester linkages between aromatic rings. The peak at 1410 cm^−1^ was assigned to C-C stretching in the aromatic ring, verifying the presence of aromatic structures. Additionally, an absorption at 1298 cm^−1^ was linked to the C-O stretching of ester groups, while the peak at 996 cm^−1^ indicated alkenes through the C-H bending of double bonds. Bands at 813 cm^−1^ and 662 cm^−1^ corresponded to C-Cl bonds and C-H bending in aromatic compounds, respectively. These findings align with similar absorption patterns reported by Leyton et al. [65], confirming the detailed molecular structure of PHL.

#### 3.3.2. HPLC Chromatogram Analysis

The HPLC chromatogram analysis was conducted to purify and identify PHL by comparing it to a standard PHL sample. This resulted in the detection of distinct peaks representing PHL, which were validated against the standard PHL peak (Figure 1B; standard PHL peak). The HPLC retention time analysis confirmed these peaks at 22.226 min, indicating the successful isolation and characterization of PHL from the brown seaweed *R. intricata* extract (Figure 1B; purified PHL peak). This successful purification process substantiates the presence of PHL in the extract and provides valuable insights into its composition (Figure 1B). In our study, the HPLC analysis of PHL showed a retention time of 22.226 min, which differs from those found by Leyton et al. [65], who reported 34.4 min, and Hasan et al. [66], who reported 3.215 min. Discrepancies in retention times can arise from variations in analytical conditions such as the type of column used, the constituents of the mobile phase, and the methods of sample preparation. Although retention time serves as a reference for compound identification, further confirmation through techniques like mass spectrometry is essential. Despite the differences, both studies highlight the importance of method validation and underscore the need for collaboration within the scientific community to enhance the understanding of PHL analysis by HPLC.

#### 3.3.3. NMR Analysis

The ^1^H NMR chemical shift at δ = 8.213 ppm indicates the presence of aromatic protons on a benzene ring, suggesting the existence of an aromatic ring structure. The peak at δ = 5.856 ppm likely corresponds to a phenolic hydroxyl group attached to the benzene ring, with phenolic protons resonating in this range, especially when involved in hydrogen bonding or within an aromatic system. The chemical shift at δ = 3.068 ppm indicates protons on carbons adjacent to electronegative atoms, such as in methoxy or other alkoxy groups. The peaks ranging from δ = 2.041 to δ = 2.059 ppm suggest protons in methyl groups connected to or near an aromatic ring, implying the presence of methyl groups directly attached to the benzene ring or integrated into an extended conjugated system. The ^1^H NMR data overall point to an aromatic benzene ring with substituents including phenolic hydroxyl groups and possibly methoxy or alkoxy groups, consistent with a complex phenolic compound such as a PHL derivative (Figure 1C). Karthik et al. [50] utilized ^1^H NMR to analyze PHL in D_2_O, finding aromatic proton shifts at δ = 6.5 ppm, differing from our observed shift of δ = 8.213 ppm, potentially due to solvent effects or sample variation. They identified δ = 3–6 ppm shifts for ether and ester hydrocarbons, including O-methyl protons (from δ = 3.1 to δ = 3.9 ppm), suggesting a trace mannitol presence. They also noted resolved peaks at δ = 3 and δ = 5 ppm, attributed to hydroxylic groups in polymeric subunits [50].

The provided ^13^C NMR chemical shifts offer detailed insights into the carbon environments in PHL. The resonance at δ = 207.126 ppm is indicative of carbonyl carbons (C=O), typically seen in ketones or aldehydes, suggesting the appearance of these functional groups. The shift at δ = 159.153 ppm corresponds to aromatic carbons (C=C) within benzene rings or other aromatic structures characteristic of PHL. The δ = 94.523 ppm shift is representative of aliphatic carbons (C-H), such as those found in methyl or methylene groups. Additionally, the shifts at δ = 29.548, δ = 29.393, δ = 29.240, δ = 29.086, δ = 28.931, δ = 28.773, and δ = 28.624 ppm indicate aliphatic carbons (C-H) that are commonly present in alkyl chains or side groups. These latter shifts may reflect impurities or solvent-related signals, rather than the intrinsic functional groups of purified PHL (Figure 1D). Wang et al. [67] provide a corroborative perspective on the ^13^C NMR spectra, reinforcing the structural identification of PHL. Their analysis highlights key spectral features, including resonances corresponding to keto carbons and oxygenated carbons, which align well with the aromatic carbon signals observed in the ^13^C NMR spectrum. Wang et al. [68] also identified six distinct aromatic carbon signals, which are consistent with the expected aromatic nature of PHL. This congruence between the ^1^H and ^13^C NMR data, including the identification of aromatic and oxygenated carbons, offers a robust confirmation of the PHL molecular structure and validates its composition.

#### 3.3.4. LC-MS Analysis

**Figure 2:**
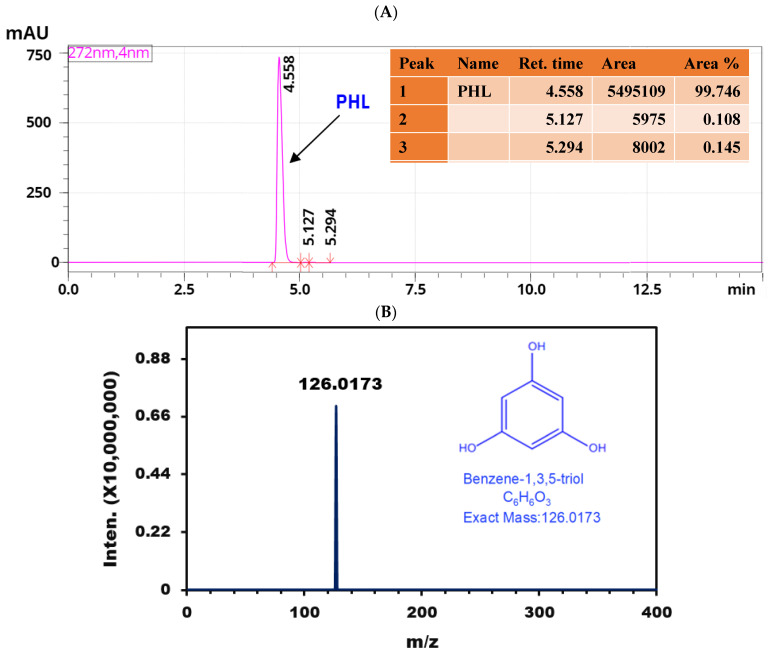
LC-MS analysis of purified PHL derived from *R. intricata*: (**A**) shows the LC chromatogram spectrum, and (**B**) presents the extracted mass spectrum of the LC peak.

The LC-ESI-QTOF-MS/MS analysis of the purified extract from *R. intricata* identified a significant quasi-molecular ion ([M + H]^+^) at *m*/*z* 126. This peak was detected at a RT of 4.558 min, indicating a molecular mass of 126.0173 Da (Figure 2A). To confirm the identity of this ion as PHL, we subjected the *m*/*z* 126 ion to MS/MS fragmentation at six different collision energies in the positive ionization mode (Figure 2B). The resulting MS/MS spectra were consistent with the identification of PHL with a chemical name of benzene-1,3,5, triol, corroborating previous findings by Lee et al. [69] and Kim and Han [70], who reported a quasi-molecular ion [M + H]^+^ at *m*/*z* 127 and a deprotonated molecule [M – H]^–^ at *m*/*z* 125 for PHL. The identification of PHL in *R. intricata* indicates the potential of this seaweed as a source of valuable phenolic compounds with possible bioactive properties. The observed quasi-molecular ion at *m*/*z* 126 closely matches established mass spectrometric characteristics of PHL, reinforcing the reliability of this identification.

### 3.4. Characterization of Synthesized ZnO NPs and ZnO-PEG-PHL NPs

#### 3.4.1. UV-Visible and Stability Analysis

**Figure 3:**
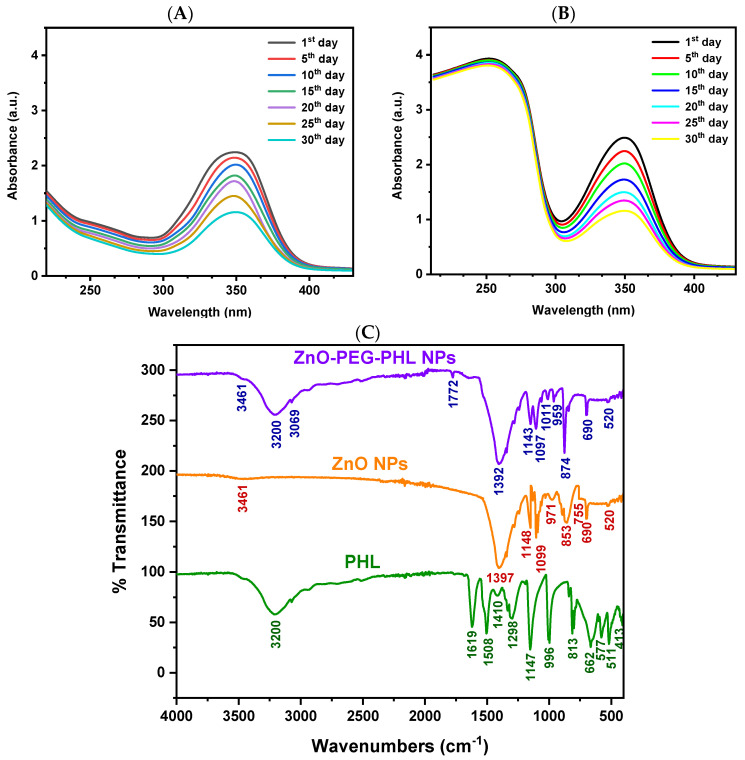
The stability analysis of synthesized ZnO NPs in the UV-visible spectrum from the 1st day to the 30th day is shown in (**A**) and that for ZnO-PEG-PHL NPs is shown in (**B**). The FTIR spectral analyses of purified PHL, synthesized ZnO NPs, and ZnO-PEG-PHL NPs are presented in (**C**).

The ultraviolet-visible absorption spectroscopic analysis revealed a distinct absorption peak at 350 nm for the synthesized ZnO NPs, confirming their successful formation. Illustrated in Figure 3A, this peak corresponds with the expected properties of ZnO NPs. Over a 30-day period, stability tests demonstrated consistent nanoparticle absorbance at various intervals (1, 5, 10, 15, 20, 25, and 30 days). This indicates that the ZnO NPs maintain their functional properties over time, underscoring their optical stability for potential applications. The successful conjugation of ZnO NPs with PEGylated PHL was verified using UV-Vis spectroscopy. Absorption peaks between 220–260 nm confirmed the integration of PEG and PHL, with PEG showing a distinct band at 190–220 nm and PHL exhibiting a peak at 240–260 nm, as illustrated in Figure 3B. These results validate the presence of both components in the nanoconjugate. Our ultraviolet-visible absorption spectroscopic analysis showed an absorption peak at 350 nm, which aligns with the 365 nm reported by Ragavendran et al. [71], confirming the characteristic optical properties of ZnO NPs. Additionally, our 30-day stability assessment, which indicated no significant changes in absorbance, aligns with that by Islam et al. [72], who found sustained catalytic activity over two weeks. These findings collectively affirm the robust stability and functional reliability of ZnO NPs, supporting their potential for diverse applications.

#### 3.4.2. FTIR Spectral Analysis

The O-H stretching vibration at 3461 cm^−1^ indicates hydroxyl groups, commonly found on metal oxide nanoparticle surfaces due to adsorbed water or surface hydroxylation. The C-O stretching at 1397 cm^−1^ points to carboxylate or other organic groups likely introduced during the synthesis. The peaks at 971, 853, 755, 690, and 520 cm^−1^ represent Zn-O stretching and bending vibrations, confirming the presence of ZnO NPs (Figure 3C; ZnO NPs peak) and their successful synthesis, as supported by Handore et al. [73] and Abdelghani et al. [74].

For the ZnO NPs PEGylated with PHL, the FTIR data revealed various functional groups: O-H stretching at 3461 cm^−1^ and 3200 cm^−1^ from hydroxyl groups in PHL and PEG; C-H stretching at 3061 cm^−1^ from aromatic rings in PHL; and C=O stretching at 1772 cm^−1^, likely from ester or carboxyl groups resulting from PEG or ZnO interactions. Peaks at 1392 cm^−1^ and 1143 cm^−1^ correspond to C-H bending and C-O stretching, indicative of PEG. C-O-C stretching at 1097 cm^−1^ confirms ether linkages in the PEG, while C-H bending and O-H bending at 1011 cm^−1^ and 959 cm^−1^ are likely from PHL. Zn-O stretching at 874 cm^−1^ and 690 cm^−1^, and Zn-O bond vibrations at 520 cm^−1^ validate the presence of ZnO within the PEG matrix (Figure 3C; ZnO-PEG-PHL NPs peak). These findings align with those of Khan et al. [39] and Badry et al. [75], confirming the successful PEGylation of ZnO NPs with PHL.

#### 3.4.3. DLS Analysis

**Figure 4:**
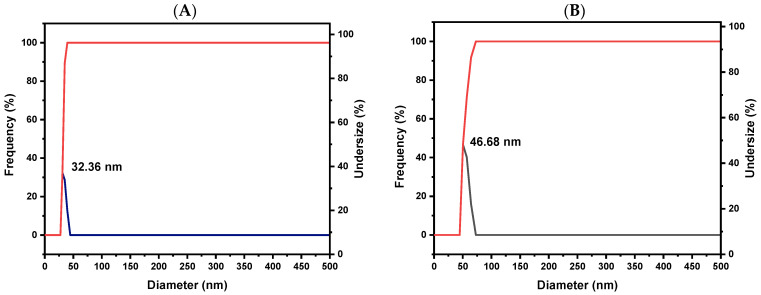

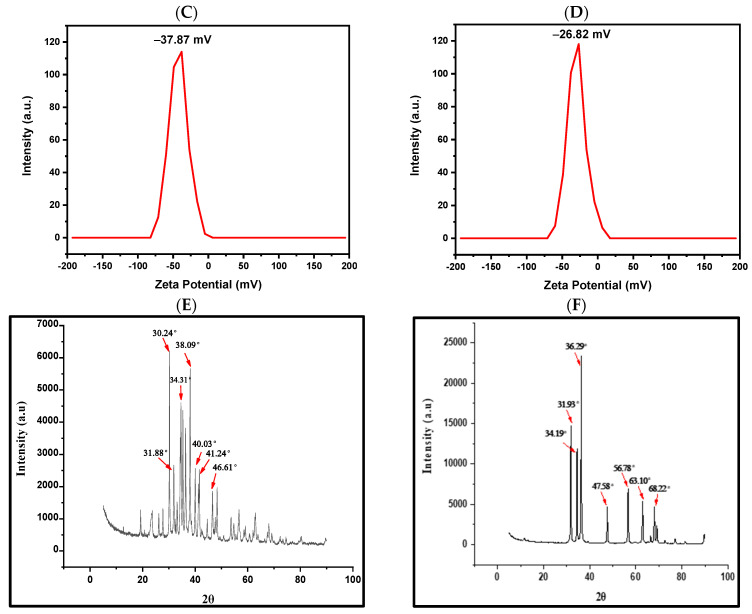
The DLS spectral analysis of synthesized ZnO NPs and ZnO-PEG-PHL NPs is shown in (**A**,**B**). The zeta sizer spectral analysis of ZnO NPs and ZnO-PEG-PHL NPs is presented in (**C**,**D**). The X-RD spectral analysis of ZnO NPs and ZnO-PEG-PHL NPs is illustrated in (**E**,**F**).

The DLS analysis confirmed an increase in the hydrodynamic diameters of the ZnO NPs upon PEGylation and the incorporation of PHL, highlighting the effective functionalization process. The observed size increase from 32.36 nm to 46.68 nm reflects the addition of the PEG and PHL layers, which is consistent with findings by Mahalakshmi and Kumar [76], who reported a similar increase in size upon functionalizing gold NPs with PHL. These results underscore the successful attachment of PHL to the ZnO NPs and provide essential insights into the size characteristics, physicochemical properties, and potential applications of the synthesized NPs (Figure 4A,B).

#### 3.4.4. Zeta Sizer Analysis

The zeta sizer analysis indicated that ZnO NPs exhibit a notable charge of −37.87 mV. Following PEGylation and the incorporation of PHL, this charge decreases to −26.82 mV. This reduction in negative charge signifies successful surface modification, which affects the stability of the NPs and their interactions with biological systems. Modifying the surface properties of nanoparticles by integrating different components is crucial for optimizing their performance across various applications. These results align with the established effects of surface modifications on nanoparticle behavior, underscoring the importance of controlled functionalization to achieve specific objectives (Figure 4C,D). Negatively charged ZnO NPs conjugated with PEGylated PHL interact with A549 cancer cells through several mechanisms, enhancing the cellular uptake via endocytosis, inducing apoptosis through ROS, disrupting cell membranes, and potentially modulating immune responses through cytokine release and increased phagocytosis [19]. Mahalakshmi et al. [76] found that Au NP conjugates exhibited a zeta potential of −31.3 mV, compared to −30.7 mV for Au NPs, indicating enhanced electrostatic stabilization due to PHL’s contribution to the surface charge density. This comparison underscores the importance of surface modification in optimizing nanoparticle properties for specific applications.

#### 3.4.5. XRD Analysis

The XRD analysis of ZnO NPs, shown in Figure 4E, displayed significant peaks at 2θ angles of 30.24°, 31.88°, 34.31°, 38.09°, 40.03°, 41.24°, and 46.61°. These peaks are indicative of the crystalline structure of ZnO NPs, reflecting well-organized molecular formations. The corresponding crystallite sizes were calculated to be 11.24, 7.57, 13, 10.9, 9.68, 11, and 9.2 nm, respectively, further validating the crystallinity of these ZnO NPs (Figure 4E). The XRD investigation of the ZnO-PEG-PHL NPs revealed peaks at 2θ angles of 31.93°, 34.19°, 36.29°, 56.78°, 63.10°, and 68.22°, indicating their crystalline properties. The calculated crystallite sizes for these peaks were 90.9 nm, 107.1 nm, 96.8 nm, 115 nm, 128 nm, and 144.2 nm, respectively, confirming the well-defined molecular arrangement within the ZnO-PEG-PHL NPs (Figure 4F). These analyses highlight the crystallinity of both ZnO NPs and ZnO-PEG-PHL NPs, with the former exhibiting crystallite sizes ranging from 7.57 nm to 13 nm, and the latter exhibiting sizes from 90.9 nm to 144.2 nm. These structural attributes suggest the potential applications of these NPs in fields requiring precise molecular organization and stability. In a similar vein, Mahalakshmi and Kumar et al. [76] demonstrated the crystalline structure of gold-nano conjugates through XRD, identifying a face-centered cubic phase with lattice planes at 2θ values of 38.13°, 45.29°, 63.68°, and 77.48°. The strong diffraction patterns found with multiple Bragg reflections confirm the crystalline nature of metallic gold, supporting its use in various applications. Both studies underscore the utility of XRD in elucidating the crystalline structures of NPs for diverse technological applications.

#### 3.4.6. SEM with EDAX Analysis

**Figure 5:**
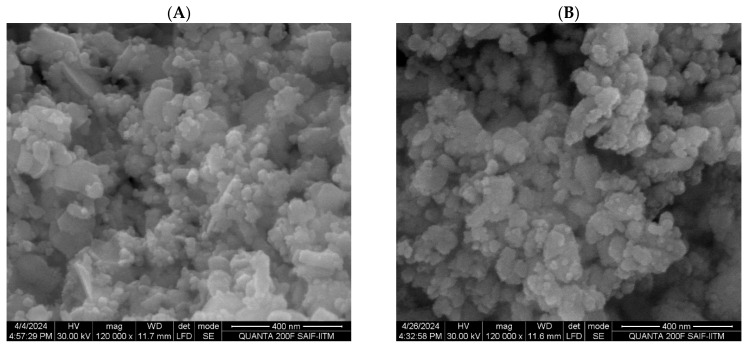

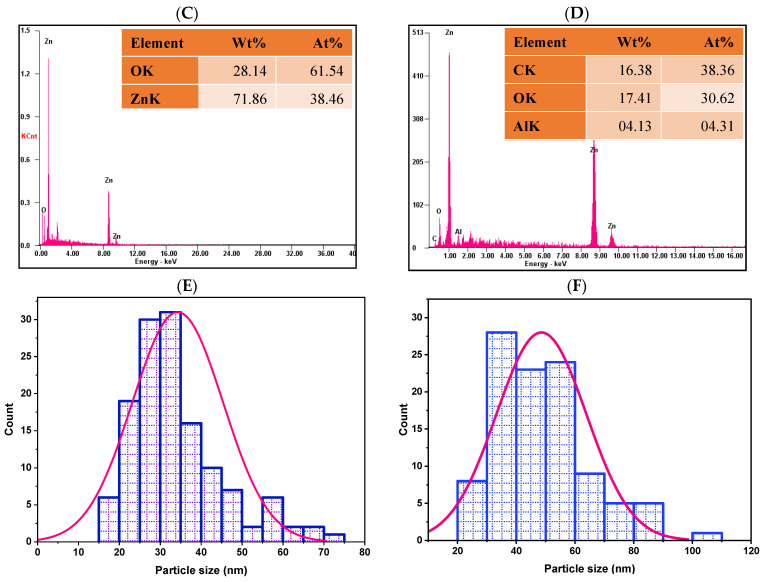
HR-SEM analysis of synthesized ZnO NPs (**A**) and ZnO-PEG-PHL NPs (**B**). HR-SEM combined with EDX spectroscopy of ZnO NPs (**C**) and ZnO-PEG-PHL NPs (**D**). Particle size distribution of synthesized ZnO NPs (**E**) and ZnO-PEG-PHL NPs (**F**) as presented in HR-SEM histograms.

The SEM analysis of the ZnO NPs showed a spherical morphology with sizes ranging from 17.125 nm to 70.957 nm, featuring a smooth surface and clear lattice fringes indicative of their crystalline nature (Figure 5A). The ZnO-PEG-PHL NPs also exhibited spherical structures, with particle sizes between 20 nm and 150 nm and a uniform morphology (Figure 5B). EDAX analysis revealed that the ZnO NPs contained 28.14% oxygen and 71.86% zinc by weight, corresponding to atomic percentages of 61.54% and 34.46%, respectively (Figure 5C). The ZnO-PEG-PHL NPs contained carbon (16.38%), oxygen (17.41%), aluminum (4.13%), and zinc (62.08%) by weight, with atomic percentages of 61.54%, 38.36%, 30.62%, and 26.71% (Figure 5D). Particle size distribution histograms indicated average sizes of 32 nm for the ZnO NPs (ranging from 17 nm to 70 nm) and 46.40 nm for the ZnO-PEG-PHL NPs (ranging from 20 nm to 150 nm) (Figure 5E,F). The spherical shape and size distribution of both types of NPs suggest well-organized particles suitable for precise applications. The average particle size of the ZnO NPs aligns with the findings by Akpomie et al. [53] and Soni et al. [77], validating the synthesis method. The porous structure of ZnO NPs further underscores their potential for various functional applications.

### 3.5. EE and LC of ZnO-PEG-PHL NPs

**Figure 6:**
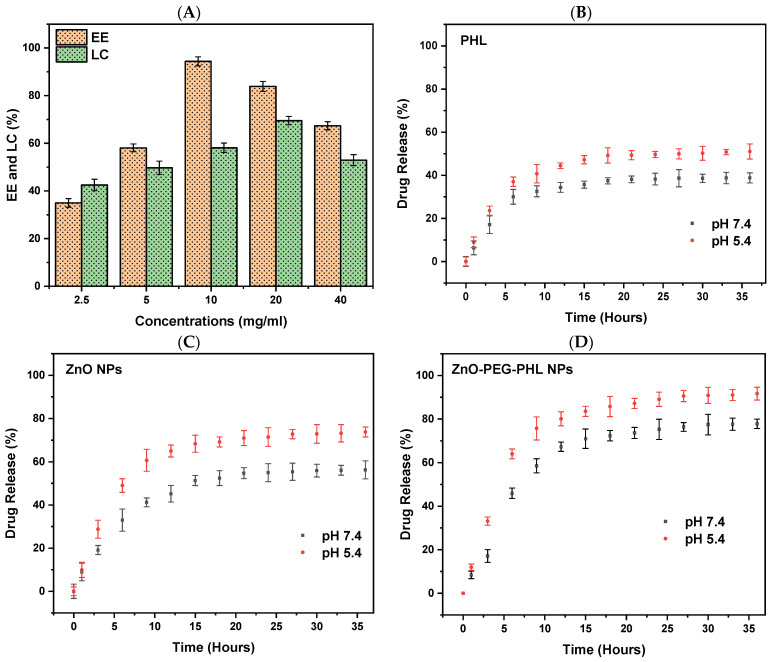
Encapsulation efficiency and drug loading capacity of ZnO-PEG-PHL NPs (**A**). Drug release percentages at pH 5.4 and 7.4 for PHL (**B**), ZnO NPs (**C**), and ZnO-PEG-PHL NPs (**D**).

The EE and LC of the ZnO-PEG-PHL NPs ranged from 34.96% to 94.37% and 42.48% to 69.51%, respectively. Among the different concentrations of ZnO-PEG-PHL NPs, the formulation with 10 mg/mL of the drug demonstrated the highest encapsulation efficiency at 94.37% and the highest loading capacity at 69.51% when the concentration was 20 mg/mL, surpassing those at 2.5 mg/mL and 40 mg/mL. Additionally, the data indicated a positive correlation between increasing concentrations of free ZnO-PEG-PHL NPs and a higher drug loading content, although this was accompanied by a reduction in encapsulation efficiency, as depicted in Figure 6A. The variation in the encapsulation and loading efficiencies of the ZnO-PEG-PHL NPs reflects the interplay between the nanoparticle concentration and drug entrapment. The optimal encapsulation efficiency at 10 mg/mL suggests efficient drug encapsulation, while higher concentrations enhance the drug loading but reduce the encapsulation efficiency. Balancing these factors is crucial for maximizing nanoparticles’ efficacy in drug delivery systems. Bacchu et al. [78] observed that ceftizoxime achieved the highest drug loading efficiency at pH 3.5, which is attributed to optimal interaction between ceftizoxime’s amine group and L-cysteine’s carboxylic group. This pH maximizes the charge difference, enhancing the adsorption, while higher pH levels increase the electrostatic repulsion, reducing the loading efficiency. Both studies emphasize the importance of fine-tuning experimental conditions such as the pH for the ceftizoximeand nanoparticle concentration for ZnO-PEG-PHL NPs to achieve their maximum drug loading and encapsulation efficiencies. These findings underscore the critical interplay between chemical interactions and physical parameters in designing effective drug delivery systems.

### 3.6. In Vitro Drug Release Study

In our in vitro drug release study of PHL, ZnO NPs, and ZnO-PEG-PHL NPs through a dialysis membrane, a 500 mL release medium used to obtain 36 h release profiles, depicted in Figure 6B–D, illustrated a sustained release of PHL, ZnO NPs, and ZnO-PEG-PHL NPs under distinct pH environments (pH 5.4 and pH 7.4), without any burst release. Specifically, at pH 5.4, the PHL showed an initial release of 8.91%, which increased to 51.03%, whereas, at pH 7.4, the initial release was 6.33%, rising to 38.8%. For the ZnO NPs, the release at pH 5.4 started at 9.91% and reached 73.77%, while at pH 7.4, it began at 8.94% and increased to 56.28%. The cumulative release rate of the ZnO-PEG-PHL NPs at pH 5.4 was 11.93%, rising to 91.65%, and at pH 7.4, it started at 8.37% and increased to 77.76%. These results indicate that the drug release is higher in encapsulated ZnO NPs with the co-polymers PEG and PHL at pH 5.4 than it is at pH 7.4, suggesting a pH-responsive release behavior influenced by the stability variations in ZnO NPs in diverse pH settings. NPs represent a promising strategy for targeted drug delivery, offering enhanced bioavailability, tissue-specific targeting, increased stability, and prolonged drug activity [79]. ZnO NPs are particularly notable in contemporary drug delivery systems due to their facile synthesis, cost-effectiveness, biocompatibility, superior drug retention capacity, controlled release profile, and site-specific targeting capabilities [80]. These systems employ diverse stimuli to achieve the precise delivery of anticancer agents, thereby augmenting the therapeutic efficacy [19].

### 3.7. Antioxidants Assays

#### 3.7.1. DPPH Assay

The DPPH assay assesses antioxidant capacity by measuring the hydrogen donation of a substance to DPPH radicals, resulting in a color shift from dark purple to light yellow. Antioxidants such as ascorbic acid, and polyphenols facilitate electron delocalization within DPPH, decreasing its color vividness. In this study, the DPPH radical scavenging activities at 517 nm were 42.38% for PHL, 53.59% for the ZnO NPs, 82.81% for the ZnO-PEG-PHL NPs, and 88.11% for standard ascorbic acid. The highest activity being seen in the ZnO-PEG-PHL NPs is likely due to synergistic effects that enhance the electron transfer and stabilize radicals, indicating their strong potential as antioxidants. Hameed et al. [81] reported a significant antioxidant activity for algal-based ZnO NPs, with IC_50_ values of 16.1 µg/mL and 18.89 µg/mL, outperforming the algal extract alone (IC_50_ = 21.2 µg/mL). This improved activity is attributed to phenolic molecules in the algae extract, which enhance both the antioxidant activity and the ZnO NPs’ synthesis. Karkhane et al. [82] highlighted the efficacy of the DPPH scavenging assay for measuring the antioxidant activity of ZnO NPs, showing a peak activity of 58% at 45 μg/mL. Comparatively, *Sargassumvulgare*, ascorbic acid, and BHA had activities of 40%, 76%, and 80%, respectively. The green synthesis of ZnO NPs, enhanced by bioactive compounds like polyphenols and flavonoids, is more suitable for biological applications due to the resulting improved antioxidant properties [83,84].

#### 3.7.2. FRAP Assay

The FRAP assay was used to measure the antioxidant potential by evaluating the decreasing of Fe^3+^ to Fe^2+^, which forms a blue Fe^2+^-TPTZ complex detectable at 593 nm. The results showed that the PHL, the ZnO NPs, the ZnO-PEG-PHL NPs, and ascorbic acid had reductions of 38.89%, 50.71%, 75.34%, and 83.48%, respectively. The ZnO-PEG-PHL NPs demonstrated the highest antioxidant activity, which is likely attributable to the collaborative effect of their ingredients enhancing their electron donation and radical scavenging capabilities. Similarly, Khoushika Raajshree and Durairaj [85] found that ZnO NPs exhibited a higher FRAP activity (73%) compared to crude algal extract, with ascorbate as the standard (77%). This method underscores the importance of the redox potential in neutralizing free radicals. Our studies highlight the superior antioxidant capabilities of ZnO-based NPs, particularly when combined with other enhancing agents like PEG and PHL. These findings demonstrate their potential for applications requiring strong antioxidant properties.

#### 3.7.3. ABTS Assay

The ZnO-PEG-PHL NPs exhibited the highest antioxidant capacity at 69.92%, compared to the PHL (48.06%), the ZnO NPs (57.86%), and the reference standard ascorbic acid (80.16%). The superior efficacy of ZnO-PEG-PHL NPs is due to the combined effects of ZnO NPs and PEG modification, which enhance their stability and dispersibility, leading to the better scavenging of ABTS+ radicals (Figure 6). This suggests significant potential for ZnO-PEG-PHL NPs in pharmaceutical and food preservation applications due to their robust antioxidant properties. Similarly, Aydin Acar et al. [86] found that *Calendulaofficinalis*-mediated ZnO NPs at 1000 μg/ml inhibited ABTS radicals by 46.63%. Dianati et al. [87] reported a 21.033% antioxidant activity for curcumin-mediated ZnO NPs at 500 μg/mL. Ihsan et al. [88] noted 46.05% and 42.79% ABTS activities for *Curcumazedoaria*- and *Momordicacharantia*-mediated ZnO NPs at 1000 μg/mL, respectively. These variations in antioxidant activity can be associated with differences in nanoparticle size and surface area influenced by the plant extracts used in the synthesis process.

#### 3.7.4. RP Assay

The RP assay of PHL, ZnO NPs, and ZnO-PEG-PHL NPs was analyzed for antioxidant activity, with ascorbic acid as the standard reference. This method evaluates the electron-donating capacities of antioxidants, converting radical species into non-radical forms and inducing a color change from yellow to blue, which is measured spectrophotometrically at 700 nm. The results indicated antioxidant capacities of 36.56% for PHL, 47.32% for the ZnO NPs, 70.94% for the ZnO-PEG-PHL NPs, and 90.77% for ascorbic acid (Figure 6). The ZnO-PEG-PHL NPs exhibited the highest antioxidant activity, highlighting their superior effectiveness in radical neutralization, likely due to synergistic interactions between PHL and nanoparticle surface modifications. In parallel, Minhas et al. [89] employed a reducing power assay to investigate the antioxidant species in biosynthesized ZnO NPs, observing a significant reducing power of 49% at 200 µg/mL concentration. Both studies underscore the potential of Nostoc cyanobacterial extracts to effectively reduce and stabilize ZnO NPs, thereby contributing diverse antioxidant compounds to enhance their activity.

### 3.8. Cell Viability

**Figure 7:**
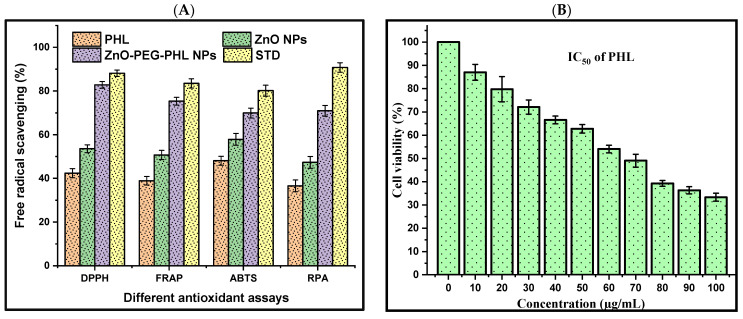

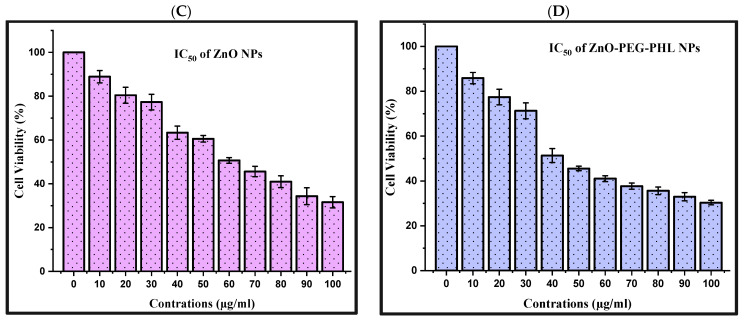
The different antioxidant activities of PHL, ZnO NPs, and ZnO-PEG-PHL NPs (**A**). The cell viability assay of A549 cancer cell line. The IC_50_ value of PHL (**B**), ZnO NPs (**C**), and ZnO-PEG-PHL NPs (**D**).

The MTT assay unveiled dose-dependent reductions in human A549 cell viability following 48 h exposures to PHL, ZnO NPs, and ZnO-PEG-PHL NPs. The viability percentages ranged from 87.02% to 33.33% for the PHL (IC_50_ = 70 µg/mL) (Figure 7B), from 88.89% to 31.54% for the ZnO NPs (IC_50_ = 60 µg/mL) (Figure 7C), and from 85.89% to 30.33% for the ZnO-PEG-PHL NPs (IC_50_ = 40 µg/mL) (Figure 7D). These findings underscore their potential as anticancer agents, suggesting mechanisms involving mitochondrial dysfunction, DNA damage, and ROS generation. Further investigations are warranted to fully elucidate their therapeutic efficacy and underlying mechanisms in cancer treatment. In alignment with these findings, Prakashkumar et al. [90] found that exposure to 35.16 μg/mL of ZnO NPs reduced cancer cell viability, showing a dose-dependent anticancer effect. Similar outcomes were observed by Nilavukkarasi et al. [91] in A549 cells, where higher concentrations induced cell shrinkage and detachment. In A-431 skin cancer cells, the IC_50_ value for ZnO NPs was determined to be 409.7 μg/mL, aligning with previous findings by Lingaraju et al. [92] for A549 cells, where the IC_50_ was reported as 383.05 μg/mL. Mohamad Sukri et al. [93] found that, unlike pure ZnO NPs, ZnO-Ag-NPs completely halted cell growth at a low concentration of 31.25 μg/mL. The MTT assay showed robust cytotoxic effects of PHL, ZnO NPs, and ZnO-PEG-PHL NPs on A549 cells, suggesting their potential as potent anticancer agents. Mechanistic studies have indicated that mitochondrial dysfunction, DNA damage, and ROS generation are involved, highlighting diverse pathways contributing to cancer cell death [19].

### 3.9. Apoptosis

**Figure 8:**
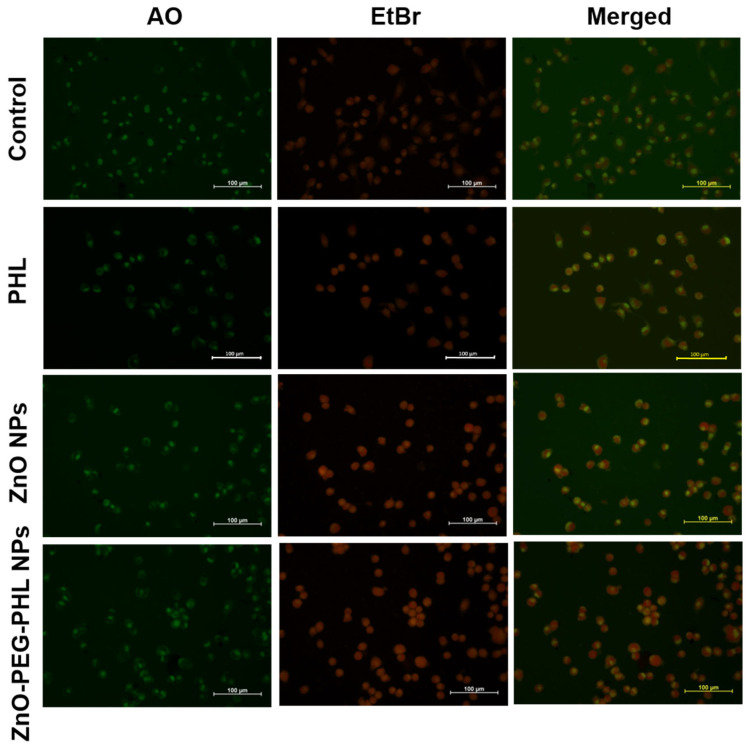
The PHL, ZnO NP, and ZnO-PEG-PHL NP substances induced programmed cell death (apoptosis) in A549 lung cancer cells following 48 h of exposure.

In this study, we used AO/EtBr dual staining to assess the apoptosis in A549 cells treated with PHL (70 µg/mL), ZnO NPs (60 µg/mL), and ZnO-PEG-PHL NPs (40 µg/mL) over 48 h. After treatment, the cells were stained with AO and EtBr and examined under a fluorescence microscope. Viable cells showed green fluorescence and intact morphology. Early apoptotic cells displayed both bright green and orange fluorescence, while late apoptotic cells exhibited orange-stained chromatin, with condensed or fragmented nuclei (Figure 8). The AO/EtBr staining revealed that the PHL, ZnO NPs, and ZnO-PEG-PHL NPs induced varying degrees of apoptosis, with the ZnO-PEG-PHL NPs showing the most pronounced effects. This staining method effectively differentiated apoptotic stages and confirmed the cytotoxic potential of the treatments, as evidenced by pyknosis, chromatin compaction condensation, and karyorrhexis [94]. Our findings align with those of Ravilla et al. [95], where Nar-ZnO NPs significantly reduced A549 cell viability, demonstrating a dose-dependent LDH release and apoptosis. Naringin-loaded ZnO NPs increased apoptotic and necrotic markers, supporting their cytotoxicity in cancer cells. Overall, this study highlights the varying apoptotic effects of these NPs and their potential for therapeutic applications.

### 3.10. Cell Cycle Distribution Analysis

**Figure 9:**
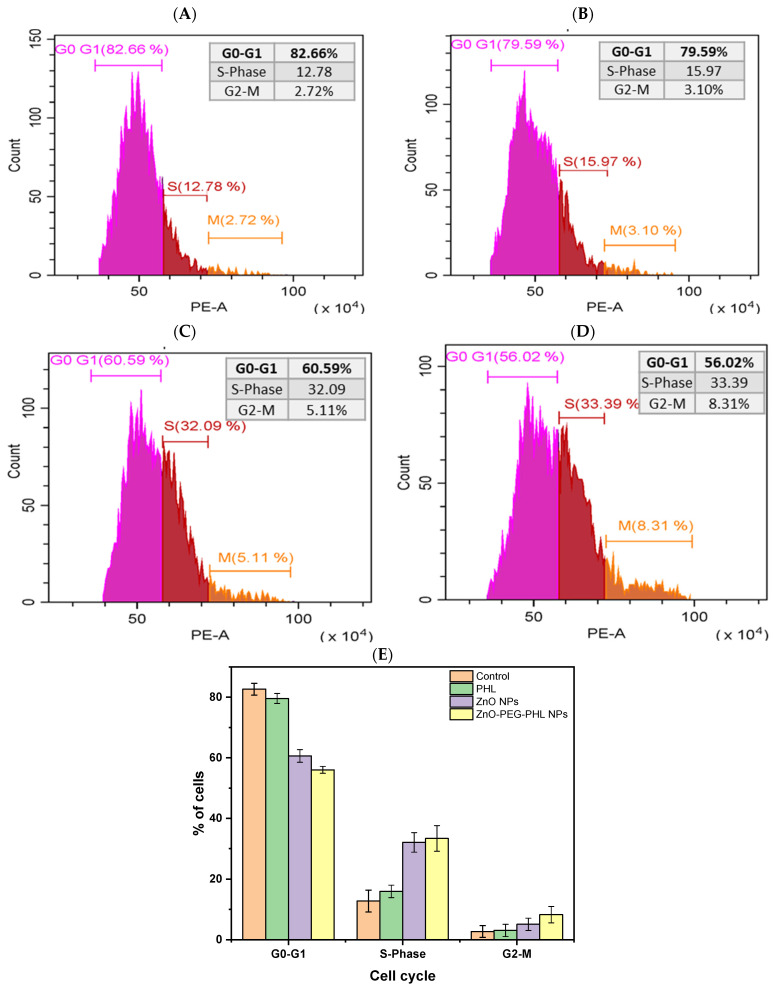
The image illustrates the effects of PHL, ZnO NPs, and ZnO-PEG-PHL NPs on the FACS activity in A549 cells for 48 h. It includes histograms showing the distribution of the cell cycle for the control group (**A**), A549 cells treated with PHL (**B**), those treated with ZnO NPs (**C**), and those treated with ZnO-PEG-PHL NPs (**D**). Panel (**E**) provides a summary of the percentage of cells in each cell cycle phase of exposure to PHL, ZnO NPs, and ZnO-PEG-PHL NPs.

The cell cycle analysis of A549 cell lines treated with PHL, ZnO NPs, and ZnO-PEG-PHL NPs revealed distinct effects. In the control group, cells were distributed as follows: 82.66% in G0-G1, 12.78% in the S-phase, and 2.72% in G2-M (Figure 9A). Treatment with PHL led to a slight reduction in G0-G1 (79.59%), with an increase in the S-phase (15.97%) and a minor rise in G2-M (3.10%) (Figure 9B). The ZnO NPs significantly altered the distribution, reducing G0-G1 to 60.59%, while increasing the S-phase to 32.09% and G2-M to 5.11% (Figure 9C). The ZnO-PEG-PHL NPs further intensified these changes, reducing G0-G1 to 56.02%, and increasing the S-phase to 33.39% and G2-M to 8.31% (Figure 9D,E). These results indicate that ZnO NPs and ZnO-PEG-PHL NPs induce substantial cell cycle arrest at the S-phase, suggesting enhanced DNA synthesis stress or replication blocking. The increased S-phase and G2-M populations for the ZnO-PEG-PHL NPs point to a more pronounced disruption in cell cycle progression, likely due to the synergistic effects of ZnO NPs and PEGylation with PHL, which may enhance their anticancer efficacy by interfering with cell proliferation. Consistent with Gao et al. [96], the ZnO NPs appear to activate the G2/M checkpoint, hindering mitosis and allowing time for DNA repair or inducing apoptosis if the damage is incurable. Our findings align with those of Pearce and Humphrey [97], showing cell cycle arrest at the S-phase and G2/M phases, leading to apoptosis.

## 4. Conclusions

This research comprehensively detailed the extraction, purification, and characterization of PHL from *R. intricata* algae. Using acetone extraction and Sephadex LH-20 column chromatography, we isolated a highly enriched fraction (F-3) containing phenolic and PHL compounds. Analytical techniques including FTIR, HPLC, NMR, LC-MS, and XRD confirmed the PHL’s identity, structure, and purity. The functionalization of ZnO NPs with PHL and PEGylation produced ZnO-PEG-PHL NPs, which were characterized by UV-Vis, FTIR, DLS, SEM-EDAX, and XRD analyses, highlighting their crystalline nature, morphology, and surface chemistry. Antioxidant assays demonstrated significant scavenging activities, with potential biomedical applications. In vitro studies revealed cytotoxic effects on A549 cells, with ZnO-PEG-PHL NPs showing potent apoptotic induction and cell cycle alteration, suggesting their potential as multifunctional agents in oncology and nanomedicine.

## Figures and Tables

**Table 1:** The yield, total phenolic content, and total phlorotannin content obtained from Sephadex LH-20 column chromatography of *R. intricata*.

Sample	Yield (%)	TPC (GAE mg/g)	TPhC (PGE mg/g)
Crude	50.03	18.09	24.09
Partially purified	30.61	21.03	30.91
Purified	13.1	32.47	45.65

TPC—total phenolic content; GAE—gallic acid equivalent; TPhC—total phlorotannin content; PGE—phloroglucinol equivalent.

## Data Availability

The data provided in this study are not openly available because of the proprietary nature of drug development.
